# Emil du Bois-Reymond and Dom Pedro II: Research development on bioelectricity and electric fish

**DOI:** 10.3389/fphys.2022.985473

**Published:** 2022-12-05

**Authors:** Roberto Pereira Santos, Antonio Egídio Nardi, Marleide da Mota Gomes

**Affiliations:** ^1^ Service of Neurology, Clementino Fraga Filho University Hospital, Federal University of Rio de Janeiro, Rio de Janeiro, Brazil; ^2^ Laboratory of History of Psychiatry, Neurology and Mental Health, Institute of Psychiatry, Institute of Neurology, Federal University of Rio de Janeiro, Rio de Janeiro, Brazil

**Keywords:** Dom Pedro II, Du Bois-Reymond, electrophysiology, history of neuroscience, history of medicine

## Introduction

Emile Du Bois-Reymond pioneered scientific electrophysiology in parallel with 19th-century studies on various electric fish that underlie the great development of modern electrophysiology (Piccolino, 1997; Piccolino, 2011).

Dom Pedro II, Brazilian Emperor, on his second long trip abroad, from March 1876 to September 1877, on his stop in Germany, met the researcher when the ruler visited the new Institute of Physiology at the University of Berlin (Bezerra, 2009; Ministério Da Educação Esaúde, 1942, p. 21–22). Shortly after (1880), with the support of Pedro II, the Laboratory of Physiology of the National Museum in Brazil was founded, directed by Louis Couty (1854–1884), a student of Claude Bernard and Alfred Vulpian, and João Batista de Lacerda (1846–1915) (Quintanilha and Murasse, 2004).

**FIGURE 1:**
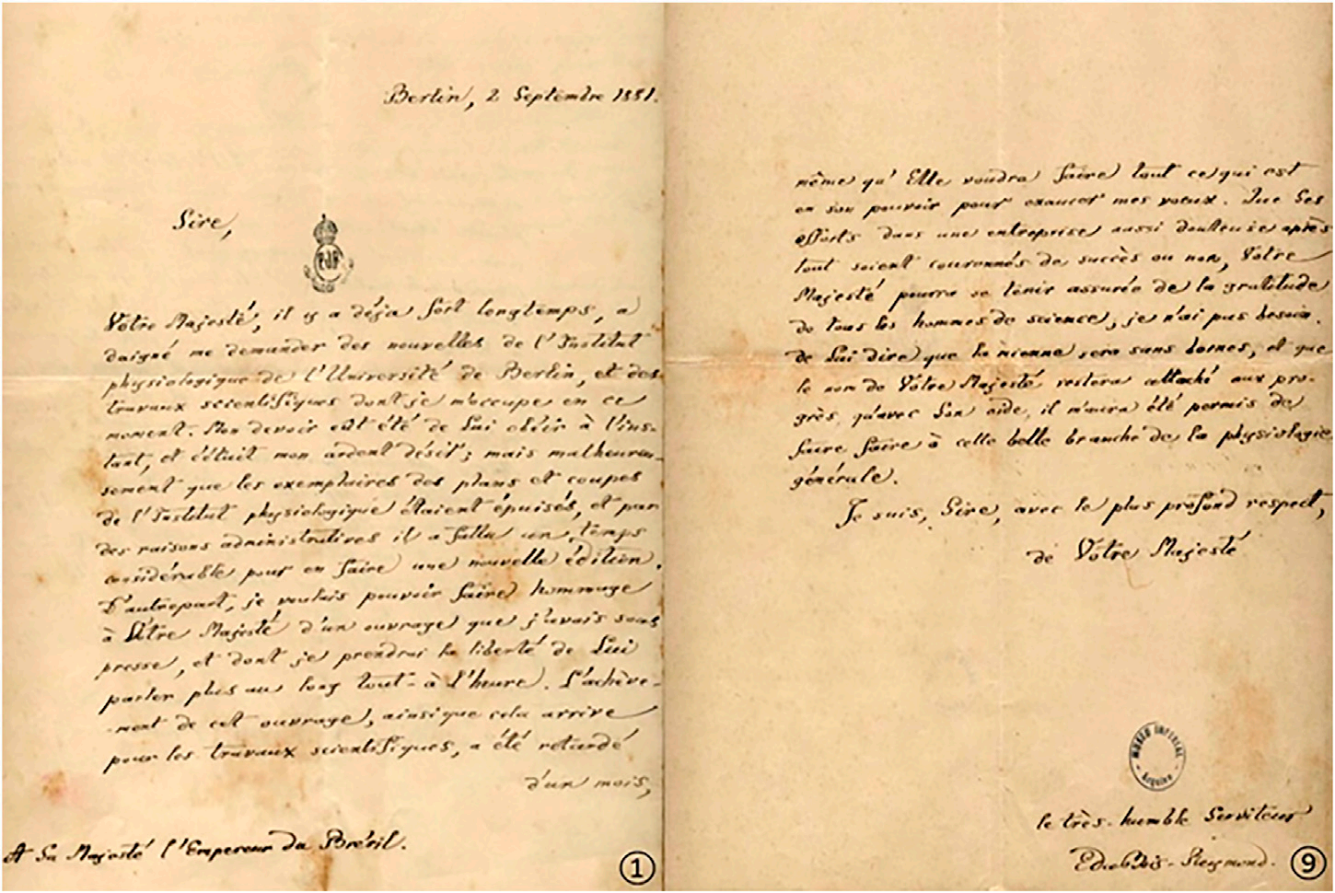
A letter from Bois-Reymond to Dom Pedro II, in 1881, about electric fish and his interest in contributing to the development of the Physiology Laboratory of the National Museum in Brazil. Emile Du Bois-Reymond, German physician and physiologist (*7 November 1818, Berlin-†26 December 1896, Berlin, aged 78). Dom Pedro de Alcântara Bragança e Habsburgo (Rio de *Janeiro, 2 December 1825—Paris, 5 December 1891, at the age of 66), the second and last Brazilian emperor, was a correspondent and partner of several international scientific institutions, interacting with influential scientists of his time in his own or state interest (Gomes, 2021). Authorized reproduction by Museu Imperial/Ibram/Ministério do Turismo/no 02/2022 (Bois-Reymond, 1881).

Concerning Bois-Reymond, his experimental work with electric fish illustrates how an exotic organism is inserted in the production of neuroscience knowledge, also illustrated here through a letter written by him to the Emperor (Figure 1).

### Emile Bois-Reymond and his correspondences with Pedro II ´

Bois-Reymond was born to a wealthy and upper-class protestant family whose maternal origins come from French Huguenot and his father from Neuchâtel, Switzerland (Burdon-Sanderson, 1897, p.230–231; Finkelstein, 2013, p.10–93). In 1836, he entered the University of Berlin with an initial penchant for the study of theology after chemistry, natural philosophy, mathematics and geology. However, he turned to the study of medicine and came to Johannes Peter Müller (1801–1858), professor of physiology, comparative anatomy and embryology at the University of Berlin, who made Bois-Reymond his assistant, in 1840, and Müller encouraged him to study nerves. While working at the University of Berlin (1836–1896), Bois-Reymond chose the theme “Electric Fish”, a historical-literary approach, for his graduation thesis (1843), which marked the beginning of a long career in bioelectricity studies (Finkelstein, 2013, p.10–93).

The observations of Carlo Matteucci (1811–1868) are considered the starting point for Bois-Reymond’s investigation into the phenomena of animal electricity, on an electrophysiological basis. With Müller’s death in 1858, the former chair of anatomy and physiology was divided into one of human and comparative anatomy and another of physiology, the latter being succeeded by Bois-Reymond (Burdon-Sanderson, 1897, p.230–231), although he rejected the vitalist views of his mentor. Along with his colleague and friend Hermann Ludwig Ferdinand von Helmholtz (1821–1894) and others, he sought to explain biological processes solely based on physicochemical principles.

Bois-Reymond and his friend Helmholtz, who was also a Müller´s pupil, were prominent personalities in Berlin. They both used their position and influence on the advancement of science. In this way, Bois-Reymond built up a new branch of science, the scientific electrophysiology.

In 1877, through Bois-Reymond’s ´influence, the government provided the university with a proper physiological laboratory “which has made the Berlin laboratory a model for similar institutions in all parts of the world”, *apud* Burdon-Sanderson (Burdon-Sanderson, 1897,p.230–231). In this same year, Pedro II met the researcher, but in 1881, they were still in touch, when Bois-Reymond sent a letter to Dom Pedro II regarding his new scientific works in Berlin:

“Your Majesty, a long time ago, deigned to ask me for news of the Physiological Institute of the University of Berlin, and of the scientific work with which I am engaged at the moment… On the other hand, I wanted to be able to pay homage to Your Majesty with a work that I had in the press, and of which I will take the liberty of speaking to You at greater length later on…”

Much of Bois-Reymond’s research was directed towards electrical phenomena thought to be involved in various life processes since Galvani’s time, but which were known to be related with certainty only to electrical discharges from fish (encyclopedia.com), those that are analogous to the potentials that arise in nerve and muscle during activity.

Many articles by Bois-Reymond, particularly after 1877, were written by his pupils such as Karl Sachs (1853–1878) and Gustav Theodor Fritsch (1838–1927), and deal with anatomy and the production of electricity in electric fishes. Beginning in 1857, he studied living examples of *Malapterurus electricus* (electric catfish), the torpedo, and *Gymnotus electricus* (Encyclopedia, 2022). The most famous production of this partnership concerns a monograph on the electric eel by Karl Sachs and edited by (Bois-Reymond, 1881): *Untersuchungen am Zitteraal: gymnotus electricus* (*Studies on the electric eel: gymnotus electricus*).

In 1881, Bois-Reymond was still working on electrical fish (Burdon-Sanderson, 1897, p.230–231), studies about electricity, and he would like to speak to Dom Pedro II about it. Furthermore, in this letter, Bois-Reymond describes a detailed origin history of his relationship with electrical fish studies and the untimely Sachs death.

“Your Majesty is aware that I have devoted my life to the study of Animal Electricity. In my youth, my strongest desire would have been to go and continue in the Llanos of Venezuela the work of Alexander of Humboldt and, having arrived in a position which would make it very difficult for me, I wanted, at least, as much as he was in my power, to favor the execution, by others, of my old projects. I had a good fortune, in 1876, to meet a young scholar, Dr Oh. Sachs, meeting the conditions necessary to be entrusted with such an enterprise, and Dr Sachs, an affect, stayed at Calabozo during the “Verano” of 1876–77 and made a host of observations on the Electric Gymnote, any kind of all-important. He was back in Europe and in the process of writing these observations, when he perished horribly, in a pleasure excursion in the Tyrolean Alps. I was compelled, unless I entirely renounced the fruits of Dr Sachs' expedition, to devote myself to the editing and elaboration of his results. It is to this that I have devoted all my leisure and all my strength for two years, and this is the origin of the beautiful volume, adorned with eight plates, to which I dare to draw in advance the benevolent attention of Your Majesty.

I did not confine myself, in this work, to a simple exposition of the new facts collected by Bachs, but I strove to give a Monograph, as complete as possible, of the most formidable of electric fishes. I have necessarily, in this continuation, encountered many points which, for the moment, remain more or less obscure. Among their number, there are undoubtedly many that, for their elucidation, require a new expedition in the regions inhabited by the Gymnotes. But there are other questions, and more important ones, which it would be easy to decide by the aid of experiments and observations made on living Gymnotes brought into Europe.”

In this letter, Bois-Reymond talks about Electric Gymnotes and their probable distribution along with Brazilian territory, asking Dom Pedro II about this electrical fish to deliver some specimens to his laboratory in Berlin.

“It is almost certain that this fish is found in all the tributaries of the Amazon and the Para; ... I dare to address myself to You, Sire, to beg Your Majesty to kindly lend me his nascent assistance by giving the necessary orders to achieve me living Gymnotes.”

More accurately, Bois-Reymond describes what is the feed and habits of Electrical Gymnotes, and how transport to Germany needs to be. There is no clear data if these requirements were accomplished.

“A very essential remark is this: as it is important to me to be able to sacrifice several gymnotes without hesitation, and not to keep some alive as objects of curiosity for the public, it would not be necessary for the consignment to consist of one or more huge fish, but like much small fish as possible, i.e. 50–75 cm at most in length. Specimens 30–40 cm in length would do just fine for me for certain experiments, at this time the most important of all.”

The former students of Bois-Reymond, Ludimar Hermann (1838–1914) and Julius Bernstein (1839–1917) added new achievements regarding “Local Circuit Theory” and “Membrane Theory”, respectively (Picollini -Galvani). This is the expression of a potential difference between the internal and external compartments of the muscle, recognized many years later with the development of Bernstein’s membrane theory that was unfolded by British scientists who recognized the chemical substances in charge of the transfer of information between the nerves and muscles (Picollino-Galvani). As for Hermann, he drew attention to the fundamental fact that the signal propagating along a nerve fiber consisted of negativity of the outer surface of the nerve, and that at the same time the fiber was stimulated when its outer surface became more negative, thus explaining the flow of current through the nerve or muscle.

Bois-Reymond began using devices and instruments to test the properties of living matter, such as the galvanometers that measured the intensity of the electrical current. In his study of electrical conduction along nerve and muscle fibers, he found in 1843 that a stimulus applied to the electropositive surface of the nerve membrane causes a decrease in electrical potential at that point. Besides, at this “point of reduced potential”, the impulse travels along the nerve as a “wave of relative negativity.” In doing so, he was able to demonstrate that this phenomenon of “negative variation” also occurs in striated muscle and is the primary cause of muscular contraction. He unveiled the fast-changing electricity associated with muscle contraction and nerve excitation—the action potentials. He defined what he called electrotonus, the potential changes produced by an externally applied current. At the same time, Bois-Reymond also reported in detail less fluctuating electricity at wounds, injury current and potential, he made to himself. The summation of his studies was published chiefly in his work of 2 vol., *Untersuchungen über thierische Elektricität* (*Researches on Animal Electricity*), the first part of which appeared in 1848, the last in 1884 (Burdon-Sanderson, 1897; Pearce, 2001). He is also known for his views on the pathogenesis of migraine (Pearce, 1986).

Bois-Reymond, in his later years, returned to some of his original interests, delivering speeches on science, philosophy, history and literature through public lectures that made him a celebrity with his provocative speeches on politics and culture, even presenting Darwin to German students (Finkelstein, 2013). Thus, he became very influential, having been admitted to the Berlin Academy of Sciences (1851), becoming its perpetual secretary in 1867, having also been rector of the University of Berlin and also its medical faculty dean (Burdon-Sanderson, 1897, p.230–231; enciclopedia.com; Finkelstein, 2013).

### Development of European experimental physiology with a touch on that of Brazil

In the 19th century, there was an unfolding of research focused on the biological, physiological and chemical foundations of life. In the meantime, the study of “animal electricity” was developing, and Bois-Reymond replaced the idea of attributing the action of nerves and muscles to vital forces with a mechanical vision, demonstrating the electrical nature of nervous signals, consequently, creating the discipline of electrophysiology within the reorganization of the natural sciences (Finkelstein, 2015).

In Brazil, Pedro II’s travels took him to Bois-Reymond of the “Berlin School”, and to its recently inaugurated Laboratory of Physiology (1877). The emperor demonstrated continued interest in being informed about the scientist’s studies and his possible collaboration in the development of the Physiology Laboratory of the National Museum in Brazil. On the other hand, Bois-Reymond tried to obtain South American electric fish for his laboratory, as written in a letter sent to Pedro II in which he contextualizes the participation of his School in these studies.

In 1882, at the emperor’s request, Bois-Reymond sent complete and detailed data about the Berlin Institute of Physiology (Dias, 1951, p.50–66), intending to develop the Physiology Laboratory in Brazil. Related to this report, in the letter (1881) from the physiologist to the statesman, we can see Bois-Reymond’s intention to donate some of his books to this laboratory building. Although this project was not carried out, there is an official statement from the government that the Emperor received some books and drawings offered by Bois-Reymond (Araújo, 1977, p.212, 243).

“The consignment that I dare to send to Your Majesty will consist of four plans and sections of the Physiological Institute, to which I will take care to attach an Explanatory Memorandum; Two large photographs representing the facade of the building, and its view from behind; A copy of my “*Gesammelte Abhandlugen zur all gemeinen Muskel-und Nervenphysik*” in two volumes, forming a collection of my Memoirs of Physiology of Muscles and Nerves extending from 1856 to 1877; From a copy of the work mentioned above, and dealing with electric Gymnote.”

In short, Dom Pedro II and Bois-Reymond sought to boost the international transfer of technology and possibly there was a contribution from the pioneer of modern electrophysiology in the early stages of the development of Brazilian experimental science inserted in a global connection and circulation of knowledge.
